# Radiofrequency Ablation of Osteoid Osteoma of Neck of Femur: A Case Report

**DOI:** 10.31729/jnma.5804

**Published:** 2021-05-31

**Authors:** Sailendra Kumar Duwal Shrestha, Anuj Jung Rayamajhi, Prabhat Rawal, Rossu Thapa, Umash Karki, Sundar Chapagain

**Affiliations:** 1 Department of Orthopedics and Trauma, Nepal A.P.F. Hospital, Balambu, Kathmandu, Nepal; 2 Department of Anesthesiology, Critical Care and Pain Medicine, Civil Service Hospital, New Baneshwor, Kathmandu, Nepal; 3 Department of Anesthesiology, Critical Care and Pain Medicine, Nepal A.P.F. Hospital, Balambu, Kathmandu, Nepal; 4 Department of Radiology, Nepal A.P.F. Hospital, Balambu, Kathmandu, Nepal

**Keywords:** *neck of femur*, *osteoid osteoma*, *radiofrequency ablation*

## Abstract

Osteoid osteoma is the most common benign bone forming tumor characterized by a nidus surrounded by reactive sclerotic bone typically associated with nocturnal pain and most common in males less than 30 years. Diagnosis may be difficult in cases of atypical presentation, intraarticular localizations or very small size. Computed tomography guided radiofrequency ablation is one of the promising treatment methods being used with an advantage of minimal invasion, faster recovery and shorter hospitalization. We present a case of an 8-year-old boy with osteoid osteoma of neck of femur managed successfully with radiofrequency ablation. To our knowledge, this is the first case reported on radiofrequency ablation in Nepal.

## INTRODUCTION

Osteoid osteoma is the most common benign, slow growing bone forming tumor.^1^ It is characterized by a nidus formed by variably calcified meshwork of bony trabeculae and fibrous, vascular and nerve tissues.^2^ It accounts for only 3% of primary bone tumors.^3^ Despite its radiologically small size (< 1.5cm), symptoms of pain are out of proportion.^4,5^ It is common in males usually <30 years of age.^6^ Diagnosis can be delayed due to atypical presentation, intraarticular localizations or due to very small size of lesion. Late presentations can lead to muscle wasting because of disuse atrophy of affected limb in children. CT scan is one of the useful investigations used to diagnose this condition. Antiinflammatory medications have been traditionally used for pain management. Although surgery is the definitive treatment, difficulty in lesion localization and the need for extensive dissection pose a problem. Radiofrequency ablation (RFA) has been found to be a safe, fast, and reliable method of treating osteoid osteomas.^7^

## CASE REPORT

An eight-year-old male child presented with a history of pain in the left hip for past 8 months. The pain was insidious in onset, progressive and aggravated at night affecting sleep and caused difficulty in walking, squatting and playing. Within two months of onset, he developed limping and wasting of left lower limb which led to antalgic gait. There was no history of trauma, fever, pain or swelling of any other joints, chest pain, weight loss or loss of appetite, and recent urinary/ gastrointestinal or respiratory tract infections. There was no family history suggestive of hemoglobinopathies. He was under muscle strengthening physiotherapy for 6 months but his symptoms were not improving.

He came to us for further consultation with persistent pain and antalgic gait. On clinical examination, the child was cooperative and active, with normal height and weight for age. There was mild tenderness over the anterior aspect of left hip joint with normal range of motion. Numeric Rating Scale (NRS) assessment of pain was 5 at rest and 7 on walking. Neurological and musculoskeletal examination revealed intact cranial nerves with intact bilateral lower limb sensation. On motor examination, muscle wasting of left lower limb was seen with mid-thigh girth difference of 2 cm and decreased power (4/5) with normal tone and reflexes. On examining the upper limb no abnormalities were found.

**Figure 1.**
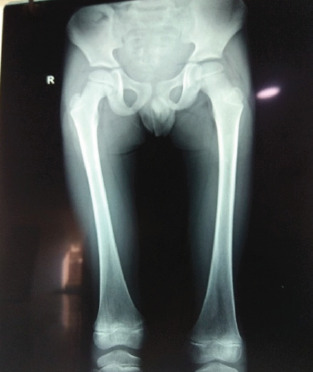
Plain radiograph showed no obvious signs of bony lesion.

Investigations showed slightly raised ESR of 12 mm/1st hour. However, the complete blood count, renal function and liver function tests, serum calcium, phosphate, vitamin D3, vitamin B12 levels were within normal range. Review of X-ray of pelvis and bilateral thighs were unremarkable (Figure 1).

**Figure 2.**
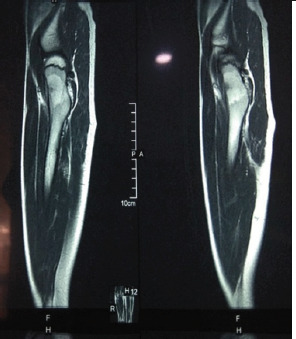
MRI showed subcortical intramedullary lesion with perilesional edema.

As suspicion was made on left hip synovitis, MRI scan of hip and thigh region was done which revealed well defined 9x8x5 mm eccentric subcortical intramedullary lesion with hypointense signal (T2w) with a hyperintense (T2w) peripheral rim in anterior half of neck of femur with perilesional marrow edema (Figure 2).

**Figure 3.**
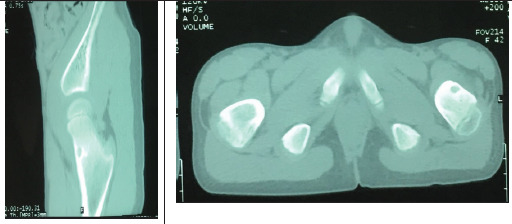
CT scan coronal and axial view showed lytic lesion with surrounding sclerotic margin over anterior aspect of left femoral neck.

Hence, a provisional diagnosis of osteoid osteoma was made and CT scan of the pelvis was further done which showed a 7x6 mm well margined lytic lesion in the anteromedial aspect of femoral neck suggestive of osteoid osteoma (Figure 3).

This led to the diagnosis of osteoid osteoma left neck of femur. Among available treatment modalities, we chose CT-guided radiofrequency ablation (RFA) of the tumor which has shown promising results circumventing the need of surgery and its resulting complications, especially, as the tumor was near the growing end of femur.^7^ The parents were given detailed explanations about the procedure and the surgical and medical alternatives available. Informed written consent was obtained.

The procedure was performed with joint effort from the Departments of Orthopedics, Anesthesiology and Radiology (Figure 4).

**Figure 4a:**
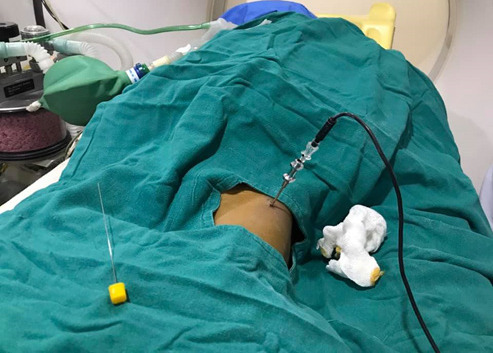
. Radiofrequency electrode introduced into osteoid osteoma nidus with the help of biopsy needle under C-arm guidance.

**Figure 4b.**
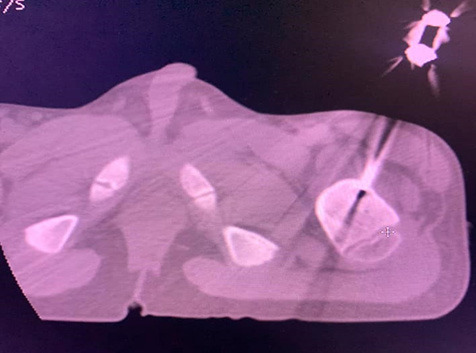
Real time CT scan axial view of left femoral neck showing osteoid osteoma nidus being ablated.

The CT scan suite was equipped with anesthesia machine and all essential drugs and resuscitation equipment required for the administration of general anesthesia. After a thorough pre-anesthetic evaluation and adequate fasting period, general anesthesia was induced on the patient with oxygen and Propofol and Laryngeal Mask Airway was inserted for airway management. Anesthesia was maintained with oxygen and inhalation anesthesia with adequate dose of Fentanyl as analgesic. The patient was positioned supine and skin preparation and draping were done as per standard protocol. Lidocaine 0.5% was infiltrated under the skin site marked for the tumor and 14-gauge bone biopsy needle was introduced into the lesion under CT guidance. The lesion was localized with the help of 16-slice multidetector-row CT scanner (Supria, HITACHI) with multiplanar evaluation to confirm accurate needle position within the nidus of osteoid osteoma. After confirmation of needle in the nidus, an 11-gauge, 12-cm long conventional Radiofrequency (RF) probe was introduced and the electrode was connected to the RF generator (HALYARD). The tip temperature of probe was set to 95°C for 4 minutes duration. Due to high impedance, the machine could not operate initially. Local anesthetic 0.5% Lidocaine 2 ml was injected through the biopsy needle cannula and after restarting the machine, two cycles of ablation were successfully performed. After the procedure, the cannula was removed and a small pressure dressing was applied at the percutaneous puncture site. Total duration of procedure was about 1 hour. Recovery of the child from anesthesia was uneventful. The next day his pain score (NRS) was assessed to be ‘zero’ both at rest and while walking. Physiotherapy was started the same day when the pain score was 0 to regain his muscle mass and for improvement of gait.

**Figure 5.**
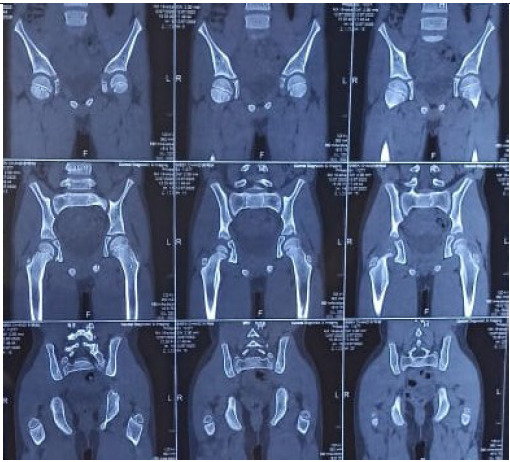
CT scan after one-year post RFA showing no residual lesion.

He was discharged from ward on the next day of the procedure with advice for follow up and prescribed simple home exercises for strengthening his thigh muscles. On follow up examination after two months, there was no complaint of pain and he gained his left thigh muscle mass with mid-thigh girth difference of 1 cm and gait was completely normal. In 12 months follow up, he was playful, without any complaint of pain and his left thigh girth was normal. His CT findings were unremarkable and no obvious residual lesion seen 12 months post RFA (Figure 5).

## DISCUSSION

Osteoid osteoma is the most common benign osteoblastic tumor of bone.^1^ Despite its relatively small size, pain is excruciating and persistent which is usually worst at night. Typically, the pain is relieved by salicylates. It generally has a self-limiting course with pain subsiding within 5 to 6 years.^8^ If the tumor is near the growing end of a bone in a child, increased blood flow may cause deformities in the form of either overgrowth or undergrowth of the limb.^9^

Diagnosis of osteoid osteoma can be done with the help of X-ray which shows radiolucent nidus surrounded by a zone of sclerosis. If there is extensive sclerosis or nidus <4 mm, nidus may not be seen in plain radiographs^10^ such as in the case described, because of its small size. It may require a thin cut CT scan which is the investigation of choice.^11^ MRI is also useful in its diagnosis which shows half-moon sign due to related bone marrow edema.^12^ Biopsy prior to treatment is not mandatory due to a remarkable amount of false negative findings in clinically and morphologically unambiguous cases of osteoid osteoma.^13^ With delayed diagnosis, features other than pain such as limping or muscle wasting and weakness can appear, which was prominent in this particular case.

In the past, treatment modalities included conservative treatment with NSAIDs, waiting for spontaneous resolution of the tumor or surgical methods including en-bloc resection, curettage of tumor or arthroscopic excision for intra-articular lesions. Surgery was the only treatment available for osteoid osteomas until recently.^9^ Difficulty in lesion localization during surgery is a major problem.^9,14,15^ Moreover, successful surgery necessitates complete removal of the tumor and thus extensive resection which causes tissue damage and may lead to structural weakening.^9,14,15^ Surgery also carries a high risk of complications and requires a long recovery period.^9,14^ Surgery for tumors in weightbearing areas also entails a long period of limited weight bearing.^9^

Other methods which are less invasive include percutaneous bone resection and drilling (PBRD)^16^ and CT-guided Radiofrequency ablation of tumor (RFA). RF ablation for treating osteoid osteoma was first described in 1989, with initial results published in 1992.^17^ RFA is the gold standard treatment for osteoid osteoma in all locations as it is an effective, safe method with lowest recovery time and rate of complications.^16^ Percutaneous RFA is a widely used interventional technique. It allows the precise delivery of heat under image guidance to the targeted tissue. It can be done in a day care basis and the symptoms are relieved immediately after the procedure. The resolution of pain is the primary parameter used to define a successful treatment. Radiographically, partial or complete infilling of the nidus with sclerotic bone is expected over 2-27 months, although little or no change in lesion appearance is also possible.^2^ So, in a nutshell, osteoid osteoma can sometimes be difficult to diagnose initially when it presents with atypical clinical features, complications or proximity of lesion to a joint. But, once diagnosed, it can be easily treated with CT-guided radiofrequency ablation (RFA) with very good clinical outcomes and an added advantage of early recovery and lesser hospital stay.

RFA is usually performed under general or spinal anesthesia. We performed RFA using general anesthesia. The procedure described above is similar to the procedure described by other authors.^2,3^ RFA is preferred over other treatment methods because it is not associated with significant complications, does not necessitate hospitalization, and requires only a short convalescence period.^18^ Difficulty in localizing the lesion, conventional method of wide dissection leading to prolonged recovery, incomplete removal and recurrence of tumor have been major issues in the conventional treatment of osteoid osteoma, which makes surgery a less preferred option at present.

RFA has proved to be a safe, quick, and minimally invasive method of osteoid osteoma treatment in many countries. We have been able to perform this procedure for the first-time in Nepal with collaborative effort from multidisciplinary faculties with a successful outcome for the patient. RFA should be considered the current method of choice for the treatment of osteoid osteomas.

## References

[1] Shankman S, Desai P, Beltran J (1997). Subperiosteal osteoid osteoma: radiographic and pathologic manifestations. Skeletal Radiol.

[2] Pinto CH, Taminiau AH, Vanderschueren GM, Hogendoorn PC, Bloem JL, Obermann WR (2002). Technical considerations in CT-guided radiofrequency thermal ablation of osteoid osteoma: tricks of the trade. AJR Am J Roentgenol.

[3] Atesok KI, Alman BA, Schemitsch EH, Peyser A, Mankin H (2011). Osteoid osteoma and osteoblastoma. J Am Acad Orthop Surg.

[4] Motamedi D, Learch TJ, Ishimitsu DN, Motamedi K, Katz MD, Brien EW (2009). Thermal ablation of osteoid osteoma: overview and step-by-step guide. Radiographics.

[5] Solomon L, Warwick D, Nayagam S (2010). Apley's system of orthopaedics and fractures.

[6] Cerase A, Priolo F (1998). Skeletal benign bone-forming lesions. Eur J Radiol.

[7] Jankharia B, Burute N (2009). Percutaneous radiofrequency ablation for osteoid osteoma: How we do it. Indian J Radiol Imaging.

[8] Parlier-Cuau C, Champsaur P, Nizard R, Hamze B, Laredo JD (1998). Percutaneous removal of osteoid osteoma. Radiol Clin North Am.

[9] Kjar RA, Powell GJ, Schilcht SM, Smith PJ, Slavin J, Choong PF (2006). Percutaneous radiofrequency ablation for osteoid osteoma: experience with a new treatment. Med J Aust.

[10] Kattapuram SV, Kushner DC, Phillips WC, Rosenthal DI (1983). Osteoid osteoma: an unusual cause of articular pain. Radiology.

[11] Hosalkar HS, Garg S, Moroz L, Pollack A, Dormans JP (2005). The diagnostic accuracy of MRI versus CT imaging for osteoid osteoma in children. Clin Orthop Relat Res.

[12] Klontzas ME, Zibis AH, Karantanas AH (2015). Osteoid osteoma of the femoral neck: use of the half-moon sign in MRI diagnosis. AJR Am J Roentgenol.

[13] Hoffmann RT, Jakobs TF, Kubisch CH, Trumm CG, Weber C, Duerr HR (2010). Radiofrequency ablation in the treatment of osteoid osteoma-5-year experience. Eur J Radiol.

[14] Barei DP, Moreau G, Scarborough MT, Neel MD (2000). Percutaneous radiofrequency ablation of osteoid osteoma. Clin Orthop Relat Res.

[15] Venbrux AC, Montague BJ, Murphy KP, Bobonis LA, Washington SB, Soltes AP (2003). Image-guided percutaneous radiofrequency ablation for osteoid osteomas. J Vasc Interv Radiol.

[16] Tillotson CL, Rosenberg AE, Rosenthal DI (1989). Controlled thermal injury of bone. Report of a percutaneous technique using radiofrequency electrode and generator. Invest Radiol.

[17] Raux S, Abelin-Genevois K, Canterino I, Chotel F, Kohler R (2014). Osteoid osteoma of the proximal femur: treatment by percutaneous bone resection and drilling (PBRD). A report of 44 cases. Orthop Traumatol Surg Res.

[18] Lee JM, Choi SH, Park HS, Lee MW, Han CJ, Choi JI (2005). Radiofrequency thermal ablation in canine femur: evaluation of coagulation necrosis reproducibility and MRI-histopathologic correlation. AJR Am J Roentgenol.

